# Success of Dental Implants Placed Without Primary Stability: A Retrospective Study in a Private Practice -

**DOI:** 10.31661/gmj.v14iSP1.4114

**Published:** 2025-12-31

**Authors:** Alireza Etezadinia, Hossein Salehivaziri, Seyed Mohammad Mirmoezzi, Emad Taghizadeh, Seyed Mohammad Mahdi Mirmohammadi

**Affiliations:** ^1^ Department of Periodontology, Shahed Dental School, Shahed University, Tehran, Iran; ^2^ Department of Clinical Education, Straumann Group North America, Andover, MA, USA; ^3^ Private Practice, Qazvin, Iran; ^4^ Department of Oral and Maxillofacial Surgery, Faculty of Dentistry, Shahed University, Tehran, Iran

**Keywords:** Dental Implants, Primary Stability, Secondary Stability, Osseointegration, Immediate Placement, Bone Grafting

## Abstract

**Background:**

This retrospective study evaluated the success rates of dental implants placed without primary stability in a private practice setting, utilizing three contemporary implant systems (Neodent GM Helix, Biotem AR, MegaGen AnyOne) and stability-oriented protocols.

**Materials and Methods:**

Records from 63 patients receiving 97 implants (Neodent GM Helix, n=35; Biotem AR, n=39; MegaGen AnyOne, n=23; 76 in maxilla, 21 in mandible) placed between January 2021 and September 2024 in Qazvin, Iran, were reviewed. Absence of primary stability was defined as insertion torque 10 N·cm and/or intraoperative spinning. Immediate placements (n=23) received grafting; all implants were submerged, uncovered at approximately 3 months, and loaded at approximately 4 months. Success was assessed using modified Albrektsson/Buser criteria. Statistical analysis included Wilson 95% confidence intervals and Fisher’s exact or χ² tests.

**Results:**

Overall success rate was 95.9% (93/97; 95% CI 89.9–98.4%). Success rate by system was 97.1 % (34/35) for Neodent, 94.9% (37/39) for Biotem, 95.7% (22/23) for MegaGen with no significant difference among the systems (P=0.91). Immediate placements achieved a 100% success rate (23 out of 23 implants), compared to 94.6% for delayed placements (70 out of 74 implants), with no significant difference (P=0.58). Success rates were 96.1% in the maxilla (73 out of 76 implants) and 95.2% in the mandible (20 out of 21 implants), with no significant difference (P=1.00). Four early failures occurred (≤4 weeks), all in delayed placements; no late failures were observed. Systemic conditions (diabetes, hypertension, hypothyroidism) did not increase failure rates (P=0.61). All implant failures occurred in posterior regions and delayed timing of placement, while anterior and immediately placed implants achieved 100% success.

**Conclusion:**

In carefully selected cases employing atraumatic techniques, socket grafting and sealing for immediate sites, and conservative loading protocols, implants placed without primary stability demonstrated clinically acceptable success rates comparable to private practice benchmarks. Nonetheless, the absence of mechanical primary stability remains a risk factor, particularly in the posterior maxilla or type IV bone; these findings do not advocate replacing primary stability. Prospective, multicenter studies with standardized Implant Stability Quotient (ISQ) monitoring are recommended.

## Introduction

Primary stability, defined as the mechanical anchorage of a dental implant in bone at place-ment, has long been considered a prerequisite for successful osseointegration, ensuring immo-bility during the critical healing phase [1][2]. Brånemark et al. established success rates of 95-98% for implants with adequate stability, linking outcomes to minimal micromotion (<100 µm) [2][3]. However, achieving primary stability is challenging in low-density bone (e.g., type IV bone), immediate extraction sites, the posterior maxilla with thin cortical layers, or anatom-ically complex regions [4][5]. Implants lacking primary stability, evidenced by spinning or in-sertion torque <10 N·cm, are at increased risk of fibrous encapsulation, as excessive micromo-tion disrupts bone-implant integration [6].

Recent advancements in implant surface engineering and surgical protocols have begun to shift emphasis from sheer mechanical anchorage toward promoting biological integration via sec-ondary stability [7]. Modern implant designs, especially those with bioactive, hydrophilic, or microtextured surfaces, are being marketed to enhance osteoblast adhesion, protein adsorption, and earlier bone formation [8][9]. For instance, controlled clinical trials have shown that bioac-tive / superhydrophilic surfaces can improve the transition from primary to secondary stability, especially in compromised bone quality [10]. Systematic reviews of low-insertion torque im-plants report high survival rates (e.g. >95 %) even with torques ≤20-35 N·cm, suggesting that mechanical deficits can in many cases be compensated by favorable biology [11]. Animal and in vivo histologic studies support that a well-designed microtopography accelerates bone appo-sition despite weaker initial anchorage [12]. Biomechanical and stability-tracking models fur-ther indicate that secondary (biologic) stability becomes the dominant contributor to overall implant stability after about 3-4 weeks of healing [13].

Immediate implant placement into fresh extraction sockets can preserve ridge contours and shorten overall treatment timelines, but it also complicates stability dynamics because the tran-sition from primary (mechanical) to secondary (biologic) stability is critical in the early weeks of healing [14][15]. Contemporary evidence shows that immediately placed implants, when proper case selection and protocols are followed, achieve high survival, with pooled 1-year sur-vival around 97%, and randomized 10-year data in the esthetic zone confirming excellent long-term outcomes [14][15]. Clinical protocols commonly pair immediate placement with grafting and socket-sealing techniques to protect the clot and support early healing, acknowledging that adequate primary stability remains a prerequisite; cases lacking sufficient mechanical stability are still considered unsuitable in most protocols [13][14].

Systemic conditions frequently seen in implant candidates can be compatible with successful osseointegration when well controlled. In diabetes, a systematic review indicates that patients with good metabolic control have survival outcomes comparable to non-diabetic individuals, though poor control increases risk [16]. Medically treated hypothyroidism is not associated with higher implant failure versus controls [17]. After myocardial infarction, elective dental surgery is typically deferred (~6 weeks after MI or bare-metal stent; longer for drug-eluting stents) and coordinated with cardiology [18]. For hypertension, a 2024 systematic review and meta-analysis found no higher odds of implant failure compared with normotensive patients, underscoring that careful medical optimization and standard surgical principles generally miti-gate risk [19].

Private practice, with its heterogeneous patients and workflow constraints, is a real-world prov-ing ground for implant protocols. Large observational series from private clinics show high survival when standard surgical principles and stability-oriented workflows are followed [20][21]. For example, a prospective case series of 4,114 SLA implants placed in two private clinics reported predictable outcomes while tracking stability with clinical testing and resonance fre-quency analysis [20]. Similarly, a multicenter study conducted in daily dental practice (non-academic settings) documented high survival and success rates for modern systems under rou-tine care [21]. At the same time, private-practice survival analyses indicate that the absence of primary stability is a significant risk factor for failure, underscoring that biologic remodeling cannot reliably compensate when initial mechanical anchorage is lacking [22].

In this study, we evaluate 97 implants (35 Neodent GM Helix, 39 BioTem AR, 23 MegaGen AnyOne) placed in a private practice in Qazvin, Iran. Because "no primary stability" is a known risk [23], our a priori hypothesis is that strict case selection, immediate placement with graft-ing, and conservative, stability-oriented protocols can still achieve survival rates comparable to routine private-practice outcomes (≥95%), while acknowledging that proceeding without pri-mary stability is generally not recommended in established guidelines. The <10 N·cm threshold (and "spinning") was chosen as a conservative operationalization of absence of primary stabil-ity, consistent with literature linking low insertion torque to increased risk of early complica-tions or failure [23][24][25][26].

## Materials and Methods

### Study Design

This retrospective study analyzed 97 dental implants placed without primary stability, defined as priori as insertion torque <10 N·cm and/or observable spinning during cover-screw place-ment, in a private dental practice in Qazvin, Iran, between January 2021 and September 2024.

Data were derived from routine care and analyzed in de-identified form. All patients provided written consent for secondary use of their anonymized records. All procedures conformed to the Declaration of Helsinki [25].

### Patient Selection

Clinical success endpoints were anchored to classic implant success frameworks [1][24]. The primary outcome was implant success, defined per modified Albrektsson/Buser criteria as no clinical mobility, no pain or suppuration/infection, and no peri-implant radiolucency on periap-ical radiographs, with the implant in function at ≥6 months after loading [1][24].

Inclusion criteria: (1) Placement of a Neodent GM Helix, BioTem AR, or MegaGen AnyOne implant; (2) no primary stability intraoperatively (defined as insertion torque <10 N·cm and/or observable spinning during cover-screw placement) [23][24][25][26]; (3) age ≥18 years; (4) complete clinical/radiographic records for ≥6 months or until failure.

Exclusion criteria: (1) Uncontrolled systemic conditions (e.g., poorly controlled diabetes, ac-tive chemotherapy), (2) heavy smoking (>10 cigarettes/day), (3) untreated periodontitis, (4) incomplete records.

A total of 63 patients (34 female, 29 male; 25-73 years) with 97 implants met criteria. Lack of primary stability was an intraoperative finding, not planned.

### Implant Characteristics

Implant systems included Neodent GM Helix (JJGC Indústria e Comércio de Materiais Dentá-rios S.A., Curitiba, Brazil), BioTem AR System (BioTem, Seoul, South Korea), and MegaGen AnyOne (MegaGen Implant Co., Ltd., Gyeongbuk, South Korea).

Neodent GM Helix (Grand Morse) is a hybrid tapered body designed for under-osteotomy and primary stability, 16° Morse-taper connection with platform switching [27][28].

BioTem AR System has internal connection with hex + Morse-taper geometry; v-shaped mac-ro-threads; bone-level design for a range of bone qualities (manufacturer specifications) [29][30].

MegaGen AnyOne has tapered, knife-thread macro-design with internal Morse-taper; calcium-incorporated SLA (CaTiO nano-layer) surface marketed to promote early apposition [31][32].

Context for stability and surfaces: ISQ serves as a clinical indicator of implant stability; ret-rospective clinical data and literature reviews suggest that bioactive or hydrophilic surfaces may accelerate the transition from primary to secondary stability, and that selected low-torque cases can still achieve high survival rates [9][33][11].

### Surgical Protocol

A single periodontist performed all surgeries under local anesthesia (2% lidocaine with 1:100,000 epinephrine; prilocaine 30 mg with felypressin 0.03 IU; or 4% articaine with 1:200,000 epinephrine). Pre-surgical planning included clinical examination, panoramic radiog-raphy, and CBCT for site assessment. Surgery was performed freehand (no guides), aligning implant positioning to prosthetic and anatomic requirements. Osteotomies followed manufac-turer guidance; under-preparation was used in low-density bone (Lekholm-Zarb type III/IV) to improve engagement [34][35].

Twenty-three immediate implants were placed into fresh extraction sockets. When a jumping gap remained, sockets received demineralized cortico-cancellous particulate (500-1000 µm) and a gelatin sponge (ROEKO Gelatamp) to seal the socket and stabilize the clot [36]. All im-plants were placed as two-stage (submerged) and sutured with resorbable or non-resorbable ma-terial. Peri-operative medications included amoxicillin 500 mg, TID for 7 days (or clindamycin 300 mg, TID for penicillin-allergic patients) and ibuprofen 400 mg PRN; note that evidence supports at least a single pre-operative dose of amoxicillin to reduce early failures, while bene-fits of extended post-operative courses are less certain [37].

### Post-surgical Care and Follow-up

Patients were instructed to rinse with 0.12-0.2% chlorhexidine gluconate twice daily for 1-2 weeks, consistent with common post-surgical protocols [38][39]. Follow-up visits were sched-uled at 2 weeks (for suture removal), 4 weeks, 6 weeks, and 3 months, with additional appoint-ments as needed for prosthetic planning. At approximately 3 months, second-stage (uncover-ing) surgery was performed. Standardized periapical radiographs were obtained to confirm the absence of peri-implant radiolucency before placement of healing abutments.

When clinical stability and healthy peri-implant tissues were evident, healing abutments were connected and soft-tissue conditioning initiated. Prosthetic loading commenced at approxi-mately 4 months, consistent with consensus recommendations for early loading in appropriate-ly selected cases [40][41].

### Study Outcome

At the ≥6-month post-loading evaluation, each implant was assessed for success, defined ac-cording to modified Albrektsson/Buser criteria [1][24]; Implants not meeting all criteria or re-moved for any reason were classified as failures:

1. Absence of clinical mobility;

2. Absence of pain, suppuration, or peri-implant infection;

3. Absence of peri-implant radiolucency on periapical radiographs; and

4. The implant remaining in functional occlusion without complication.

### Data Collection

From patient charts we extracted: demographics (age, sex); medical history (systemic condi-tions, smoking); implant details (system, diameter, length, site/position); intraoperative stabil-ity (insertion torque <10 N·cm); placement type (immediate vs delayed); jaw/region (maxil-la/mandible; anterior/posterior); and outcomes (success/failure, time-to-failure, reason for fail-ure). Failures were classified as early (<3 months post-placement) or late (≥3 months).

### Statistical Analysis

Analyses were conducted per implant. Descriptive statistics summarized continuous variables as mean ± SD and categorical variables as counts (%). Success rates were reported overall and stratified by implant system, placement type, jaw/region, and patient characteristics; 95% con-fidence intervals for proportions used the Wilson score method; The Wilson score method was used to calculate 95% confidence intervals for success proportions, providing more accurate and reliable interval estimates than the traditional Wald method, particularly for proportions near 0 or 1 and in smaller sample sizes; It produces a 95% confidence interval that reflects the range within which the true population success rate is likely to lie, with 95% confidence [42][43].

## Results

**Table:** Table1. Implant Success Rate Stratified based on Gender, System, Placement Timing, Jaw and Region, and Systemic Diseases

**Variable**	**Category**	**Total (N)**	**Success (n)**	**Failure (n)**	**Success %**	**95% CI**	**P-value**
**Overall (implants)**		97	93	4	95.88	89.87-98.38	—
**Overall (patients)**		63	60	3	95.2	86.91% - 98.37%	—
**Gender (patients)**	Female	34	33	1	97.1	85.08% - 99.48%	0.21
	Male	29	26	3	89.7	73.61% - 96.42%	
	Neodent GM Helix	35	34	1	97.14	85.47-99.49	
**Implant system**	BioTem AR	39	37	2	94.87	83.11-98.58	0.91
	MegaGen AnyOne	23	22	1	95.65	79.01-99.23	
**Placement timing**	Immediate	23	23	0	100	85.69-100	0.58
	Delayed	74	70	4	94.59	86.91-97.88	
**Jaw**	Maxilla	76	73	3	96.05	89.03-98.65	1.00
	Mandible	21	20	1	95.24	77.33-99.15	
**Region**	Anterior	27	27	0	100	87.54-100	0.28
	Posterior	70	66	4	94.29	86.21-97.76	
	Diabetes	30	28	2	93.33	78.68-98.15	
	Hypertension	35	34	1	97.14	85.47-99.49	
	Thyroid deficiency	25	25	0	100	86.68-100	
**Systemic disease (per implant)**	Prior MI	2	2	0	100	—	0.61
	Healthy	36	34	2	94.44	81.86-98.46	
	Thyroid deficiency	25	25	0	100	86.68-100	
	Prior MI	2	2	0	100	—	
	Healthy	36	34	2	94.44	81.86-98.46	

**Table:** Table2. Details of Implant Failures

**Failure # **	**Implant System **	**Tooth / Region **	**Placement**	**Patient Characteristics **	**Implant Size / Components **	**Time to Failure **
1	Neodent GM Helix	Right mandibular 1st premolar	Delayed	38F, healthy	3.75 × 10 mm; 2 mm cover screw	3 weeks
2	BioTem AR	Right maxillary 1st premolar	Delayed	63M, controlled diabetes (HbA1c 5.9%)	4.0 × 10 mm; non-micro-thread SLA	3.5 weeks
3	BioTem AR	Right maxillary 1st molar	Delayed	63M, controlled diabetes (same as #2)	5.0 × 8.5 mm; non-micro-thread SLA	3.5 weeks
4	MegaGen AnyOne	Left maxillary canine	Delayed	51M, controlled hypertension	4.0 × 10 mm; full-arch case	4 weeks

### Participant and Implant Characteristics

Sixty-three patients (34 female, 54.0%; 29 male, 46.0%; mean age 49.2 ± 12.7 years; range 25-73) received 97 implants. All patients were non-smokers. Controlled systemic conditions were present in 35 patients (55.6%): diabetes (17 patients; HbA1c < 6.5%), hypertension (21 pa-tients), thyroid deficiency (14 patients), and prior myocardial infarction (1 patient, also hyper-tensive); 28 patients (44.4%) were systemically healthy. Systemic conditions were not mutually exclusive.

Implants were distributed by system as follows: Neodent GM Helix (35/97, 36.1%), BioTem AR (39/97, 40.2%), and MegaGen AnyOne (23/97, 23.7%). Diameters ranged from 3.5 to 5.0 mm, and lengths from 8 to 13 mm. Most implants were placed in the maxilla (76/97, 78.4%) and in posterior regions (70/97, 72.2%; premolar-molar). Immediate placement with simulta-neous grafting (demineralized cortico-cancellous particulate and gelatin sponge) occurred in 23 implants (23.7%); the remaining 74 (76.3%) were delayed placements without grafting. All procedures were two-stage (submerged), and primary stability was characterized by insertion torque < 10 N·cm and/or spinning in every case. Follow-up reached > 6 months post-loading for 88 implants (90.7%) and exactly 6 months for 9 implants (9.3%).

The overall implant success rate was 95.88% (93/97 implants; Wilson 95% CI [89.87%, 98.38%]). Success rates stratified by implant system, placement timing, jaw, region, and sys-temic condition (per-implant allocation) are presented in Table-1. No statistically significant differences in failure rates were observed across gender (Fisher’s exact test, P=.21), implant systems (Fisher’s exact test, P=.91), placement timing (Fisher’s exact test, P=.58), regions (Fisher’s exact test, P=.28) jaw (Fisher’s exact test, P=1.00), or systemic condition groups (Pearson’s χ² test, P=.61). All implant failures occurred in posterior regions and delayed timing of placement, while anterior and immediately placed implants achieved 100% success.

### Failure Analysis

Four early failures occurred (all ≤ 4 weeks post-placement; no late failures). All failures were in delayed placements. Details of the failed implants are provided in Table-2. No preceding in-fection, suppuration, or patient-reported pain was documented. Two failures occurred in the same patient. No peri-implantitis or other postoperative complications were recorded, and fol-low-up compliance was 100%. Given clustering of two failures in one patient, patient-level success (counting any failure once per patient) was 95.2% (60/63 patients).

## Discussion

In this private-practice cohort of implants placed without primary stability, overall success was 95.88%, which is consistent with contemporary real-world series from private clinics when sta-bility-oriented protocols are followed (like large private-practice cohorts and multicenter daily-practice studies) [20][21]. At the same time, success analyses from private practice warn that absence of primary stability increases failure risk, a context that frames our result as encourag-ing but still protocol-dependent [22]. Mechanistically, the result aligns with the view that bio-logic (secondary) stability becomes increasingly important during early healing, provided at-raumatic surgery and stable wound conditions are maintained [14][9].

In our study, all implant failures occurred in posterior regions and delayed timing of placement, while anterior and immediately placed implants achieved 100% success. Moreira et al. reported significantly higher primary and secondary stability in the anterior maxilla compared with pos-terior regions of the jaws, consistent with our findings [47]. This difference of anterior and pos-terior success rate is attributed to lower bone density and more challenging bone conditions in posterior areas, which reduce implant stability. Similarly, a retrospective case series by Lee et al. [48] examining 169 implants with manual rotation indicative of low primary stability found cumulative survival rates of approximately 94.7% at long term follow up, reinforcing that os-seointegration is possible without conventional mechanical fixation when healing protocols are controlled, although they observed that more complex surgical factors might elevate failure risk, this echoes our report of early failures occurring predominantly in delayed placements or posterior regions. Cobo Vázquez et al. [49] conducted a cohort study analyzing 2,400 implants, 92 of which were placed without primary stability, and reported that the absence of primary stability did not significantly influence implant survival at 12 months, though there was a trend toward early loss in certain classes of instability; this aligns with your overall high success rate (95.9%)

Surface and connection design likely contributed. Our three systems employ microtex-tured/hydrophilic or bioactive surfaces and conical (Morse-taper) internal connections intended to favor early bone response and minimize microleakage; this is coherent with literature link-ing such surfaces to faster transition from primary to secondary stability [9] and with systemat-ic evidence that low-torque cases can still achieve high survival under appropriate protocols [11]. We refrain from attributing causation to a specific brand feature; rather, the comparable performance across systems in this series suggests that sound surgical and restorative control may be at least as critical as implant model selection [20][21][22].

A key finding was 100% success in immediate placements (23/23). This is compatible with pooled evidence showing high survival for immediate implants in selected indications [14][15]. Our protocol paired immediate placement with socket grafting and a gelatin sponge seal, which is supported as a means of stabilizing the clot / socket seal in alveolar ridge preservation and immediate protocols, potentially improving early stability of the wound environment [46]. By contrast, all four early failures occurred in delayed placements, echoing reports that outcomes in healed ridges still depend on local bone quality and surgical mechanics [34][22]. The posteri-or maxilla is traditionally challenging due to type IV bone and thin cortices [13]; our failures clustered in maxillary sites (3/4), consistent with that tendency. The anterior vs posterior dif-ference in our data (100% vs 94.3%) should be interpreted cautiously due to sample size, but is directionally compatible with known anatomic/biomechanical gradients (bone density, cortical support, and loading patterns) [13][20].

Systemic conditions appeared well-managed in this cohort and did not associate with poorer outcomes: diabetics showed 93.3% success, consistent with evidence that well-controlled dia-betes yields survival similar to non-diabetics [16]; hypertension showed high survival, in line with a recent systematic review/meta-analysis [19]; medically treated hypothyroidism is not considered a contraindication [17]; and routine timing/clearance after myocardial infarction was respected [18]. While our data are reassuring, larger samples with formal per-patient mod-eling would better isolate medical risk contributions.

### Clinical Implications

Within cautious indications, immediate placement with grafting and socket sealing may be a reliable approach even when intraoperative primary stability is absent or minimal—provided that atraumatic technique, wound stabilization, and conservative loading timelines are followed [14][15][40][46]. The comparable performance of the three systems sug-gests clinicians can prioritize protocol quality (atraumatic extraction, under-preparation in soft bone, soft-tissue management, and prudent loading) over brand-specific claims [9][34][20][21][22]. Prospective studies should (i) quantify early micromotion thresholds and biologic surrogates, (ii) compare socket-grafting/seal materials in immediate protocols, and (iii) test accelerated loading in strictly selected low-stability cases [1][40].

## Conclusion

In this private-practice cohort, dental implants placed without primary stability achieved an overall 95.88% success, including 100% success in immediate placements and 93.33-100% in patients with well-controlled systemic conditions. Within the constraints of careful case selec-tion, atraumatic surgery, socket grafting/sealing for immediate sites, and conservative loading (uncovering at ~3 months; loading at ~4 months), clinically acceptable outcomes were obtained despite low intraoperative torque/spinning.

These findings suggest that, in selected low-stability scenarios, predictable osseointegration is feasible when stability-oriented protocols and modern implant surfaces/connections are com-bined. However, the absence of primary stability remains a risk factor; our results should not be interpreted as a wholesale replacement for mechanical stability but rather as evidence that bio-logic/soft-tissue management and prudent loading can mitigate risk in real-world settings. Pro-spective, multi-center studies with standardized stability metrics (e.g., ISQ trajectories) are warranted to confirm generalizability and to compare biomaterials and loading timelines head-to-head.

## Conflict of Interest

One author is employed by a company that manufactures one of the implant systems evaluated in this study. The study was conducted independently, with no financial or material support from that company or other manufacturers. This employment had no role in study design, data collection, analysis, interpretation, manuscript preparation, or the decision to submit. The au-thors declare no other conflicts of interest.
